# Physiological Chemistry

**Published:** 1892-01

**Authors:** John A. Miller

**Affiliations:** Ph. D. (Berlin)


### Progress in Medical Science.

### PHYSIOLOGICAL CHEMISTRY.

CONDUCTED BY

JOHN A MILLER, Ph. D. (BERLIN).

### TUBERCULIN.

W. HUNTER (*Brit. Med. Jour.*, ii., 1891 ; *Jour. Chem. Soc.*, 60, 1283,) subjected Koch's tuberculin to an approximate analysis with the following results. In order of importance and amount, the substances present are :

1. Albumoses ; chiefly proto-albumose and deutero-albumose, along with hetero-albumose, and occasionally a trace of dysalbumose.
2. Alkaloidal substances, two of which can be obtained in the form of platino-chlorides.
3. Extractives, small in quantity and of unrecognized nature.
4. Mucin.
5. Inorganic salts.
6. Glycerol and coloring matter.

Serum albumin, globulin, and peptone are absent.

With regard to its action, the following conclusions are drawn :

1. Tuberculin owes its activity to at least three, and probably more, different substances.
2. Its remedial and inflammatory actions are connected with presence of certain of its albumoses, while its fever-producing properties are chiefly associated with substances of a non-albuminous nature.
3. The albuminoses are not lost by dialysis ; the latter are. It is thus possible to remove the substances which produce fever, while retaining those which are beneficial in their action. The fever is thus not essential to its remedial action, and the same may probably be said for the inflammation, although, under certain conditions, inflammation appears to be beneficial.
4. The remedial action consists in shrinking of the tuberculous tissues, and increased sealing, due to deep local congestions.
5. The remedial substance resists high temperatures. Its action is, however, lessened by a dry heat of 70°. Its properties are materially altered by changes occurring during its purification by dialysis, and thus its preparation is attended with difficulty.

6. The remedial substance and the other albumoses present are stated to belong to the class of "proteins," that is, albuminous substances derived from the protoplasm of the bacilli themselves, and not merely formed by the action of these bacilli on the surrounding tissues.

---

### HUMAN RESPIRATION, AIR BEING REBREATHED IN A CLOSED VESSEL.

W. Marcet (*Proc. Roy. Soc.*, 49, 103 ; *Jour. Chem. Soc.*, 60, 1270). The results obtained in the present inquiry are, viz. :

1. On rebreathing air in a closed vessel, less carbon dioxide is expired within a given time than in ordinary breathing, the volume of the air undergoing at the same time a slight reduction.

2. When fresh air is taken after rebreathing air in a closed vessel, the volumes of air breathed and the weights of carbon dioxide expired are greater than in ordinary breathing; this, however, passes off in a few minutes.

---

### ACTION OF PARAFFINIC NITRITES ON BLOOD PRESSURE.

J. T. Cash and W. R. Dunstan (*Proc. Roy. Soc.*, 49, 314 ; *Jour. Chem. Soc.*, 60, 1270). A broad summary of the action of the nitrites is as follows :

All produce a fall of blood pressure, and an accompanying acceleration of the pulse; the latter is not so marked after intravascular injection as after inhalation, and is also less marked in cats than in men. The respiration is affected temporarily during inhalation in various degrees by the different nitrites, and permanently by repeated administration of the same or different nitrites.

The activity of the nitrites, as measured by the fall in blood pressure after inhalation is, viz.:

(1) Secondary propyl ; (2) tertiary butyl; (3) secondary butyl ; (4) iso-butyl, nearly equal ; (5) tertiary ; (6) *a*-amyl ; (7) *b*-amyl, nearly equal ; (8) methyl ; (9) butyl ; (10) ethyl ; (11) prophyl. After intravascular injection, however, the order is nearly the reverse.

### UROBILIN IN VARIOUS DISEASES.

G. Hoppe-Seyler (*Virchow's Archiv*, 124, 30 ; *Jour. Chem. Soc.*, 60, 1278). The urobilin in the urine is increased by—

1. Stasis of the bile in the liver, provided bile can pass into the intestine, and diuresis occurs, as in polycholia.
2. Stagnation of the contents of the large intestine, not of the small intestine.
3. Hemorrhages into internal organs.

Approximately the normal amount is found in—

1. Pernicious anemia.
2. Leukemia and pseudoleukemia.

It is diminished in—

1. The suspension of hepatic activity that occurs in cachexia, inanition, and many forms of anemia.
2. Stasis of bile in the liver, if bile does not pass into the intestine and diuresis is produced.
3. In the course of jaundice, sometimes. The amount of fecal pigment is no gauge of the amount of urobilin in the urine.

The normal mean, of urobilin, amounts to 0.123 gram in 24 hours.

---

### CHEMICAL CONSTITUTION AND PHYSIOLOGICAL ACTION.

T. L. Brunton and J. T. Cash (*Proc. Roy. Soc.*, 49, 311 ; *Jour. Chem. Soc.*, 60, 1279). In continuation of former work, the present research is occupied with (1) the physiological action of benzene, and (2) the alterations which occur in its action when one or more hydrogen atoms are replaced by (*a*) haloid radicles, (*b*) alcohol radicles, (*c*) by hydroxyl, (*d*) by $NO_2$, and (*e*) by $NH_2$. The modifications produced by temperature were also noticed.

The action of benzene and its compounds is chiefly exerted on the spinal cord, although they act also on the cerebrum, and, to a slight extent, on nerves and muscle. The effect on muscle and nerve is to weaken them, the paralysing action being stronger on the nerve than on the muscle. Their action on the cerebrum is evidenced by lethargy, both in frogs and rats. There is increased excitability of the spinal cord, there being greater diffusion of stimuli with diminished power and definiteness of movement. Large doses, however, cause paralysis.

Haloid radicles do not modify the action of benzene to the same extent as they do that of ammonia, but they do so in somewhat the same direction. Monochlor-benzene affects the cord more than benzene, causing spasm and rapid diminution of reflexes. It also weakens the circulation, but does not seem to affect muscles, or motor nerves, more than benzene. The bromo—and iodo—compounds act more powerfully on the cerebrum, and the iodo-compound has a special tendency to paralyze motor nerves, muscles, cerebral reflexes, and to depress the heart. Heat accelerates and cold retards the action of the substances.

The substitution of alcohol radicles for the hydrogen in benzene has the effect which one would expect, in having a more sedative action on the nervous system. The circulation is little affected, and their action is more fleeting than is the case with the halogen compounds.

Substitution of hydrogen by hydroxyl increases the tendency to convulsions; this is due to action on the spinal cord.

Amido-benzene (aniline) produces symptoms resembling those caused by ammonia, consisting in a tendency to produce violent spasm and great paralysis of muscle and nerve. It differs from ammonia in the fact that the convulsions never assume the form of true tetanus.

Nitro-benzene causes lethargy, with increasing tremor or movement, and early abolition of reflex action.

The general action of these compounds on reflex time is to lengthen it, but a primary shortening was frequently observed in the case of chloro-benzene, slightly also in that of methyl-benzene, dimethyl-benzene, and ethyl-benzene.

In producing muscular rigor, chloro-benzene is more powerful than the bromo- and iodo- compounds, and is intermediate in effect between methyl-benzene and dimethyl-benzene. Of the methyl-benzene, the methyl- is the strongest, the dimethyl- next. and the trimethyl- weakest. Ethyl-benzene is of nearly the same strength as methyl-benzene in this respect.

In warm-blooded animals (cats), respiration is early affected, there being a primary acceleration followed by slowing. The heart stops before respiration in poisoning by benzene and its haloid compounds, by ethyl-benzene, amido-benzene, and nitro-benzene, whilst respiration ceases first in poisoning by the methyl-benzenes and hydroxy-benzenes.

The first effect of the benzene on the pulse and blood pressure is a quickening and rise respectively. This is followed by a slowing and a falling.

---

ACTION OF RELATED COMPOUNDS ON ANIMALS.

W. Gibbs and E. T. Reichert (*Amer. Chem. Journ.*, 13, 289; *Jour. Chem. Soc.*, 60, 1280).

*The Hydrazines.*—Phenylhydrazine hydro-chloride depresses the cerebro-spinal centers, producing, in frogs, decreased sensibility and voluntary and reflex inactivity merging into unconsciousness and paralysis. Death results from respiratory and cardiac failure, and the heart stops in diastole. In the dog, it acts at once on the heart, causing a preliminary quickening of pulse and lowering of blood pressure. The hemoglobin is changed into an abnormal form, the blood becoming almost black. Temperature falls 1-2°. Respiration is at first quickened and then slowed, either by the action of the drug on the respiratory centers, or by the deficient oxygenation of the blood. The action of ortho- and paratolylhydrazine hydro-chloride is similar, but less pronounced. Fatal dose when injected into the jugular vein of a dog, is 0.12 gram of phenyl-hydrazine, or 0.2 gram of orthotolyhydrazine, per kilo. of body weight.

*Toluylenediamine* produces in frogs general muscular depression, but does not appreciably affect the sensory and motor nerves. The heart stops in diastole. In dogs it causes muscular depression, lowering of temperature, salivation, darkening of the blood, and diminution of the rate of respiration. The heart stops in diastole, and the stomach and intestines are found to be congested after death. Small doses of 0.8 gram per kilo. injected intravenously, increase the pulse rate, blood pressure, and respiration rate; the variation in the last is caused by direct action on the respiratory centers. The fatal dose for dogs is 0.2 gram per kilo. when injected subcutaneously; much larger doses may be injected intravenously.

*Nitrophenols.*—Dinitrophenol injected into the jugular vein of a dog in small doses of 0.2 gram per kilo. causes a stimulation of the vagus, proportional to the amount injected, and a rise in pulse rate and blood pressure. With large doses the heart stops before respiration ceases, and the blood pressure falls just before death. The minimum fatal dose is 0.05 gram per kilo. injected. When given by the mouth, the respiration rate at first rises, but subsequently falls, temperature rises 2-3°, and the pulse rate first diminishes and then increases. The drug seems to act directly on the motor portion of the spinal cord. Death results from respiratory failure when the poison is slowly absorbed through the stomach, from cardiac failure, when it passes in mass into the heart. *Trinitrophenol* injected into the jugular vein of the dog stimulates the pneumogastric nerve or centers to such a degree as to cause the immediate stoppage of the heart. If the pneumogastric nerve is divided, death occurs from respiratory failure. The fatal dose is 0.06 gram per kilo.

*Nitro-benzenes.*— Nitro-benzene (or nitro-benzol) acts on the spinal cord, causing in the frog increase in reflex activity, decrease in voluntary activity, and successive increase and decrease in pulse and respiration rates. The color of the blood is changed to chocolate, the hemoglobin being converted into nitrite-hemoglobin. In the dog, when administered by the mouth, it produces salivation, unsteadiness of gait, rise of temperature and pulse rate, weakness, and unconsciousness; the respiration is quickened by a direct action on the respiratory centers. The animal may recover if the dose does not exceed 0.75 gram per kilo. When injected into the jugular vein, in which case the fatal dose is 0.15 gram per kilo., the blood pressure is diminished by the depression of the heart muscles and vaso-motor centers. The action of dinitro-benzene is similar, but more energetic.

*Amides.*—*Formamide* acts on the spinal cord, a dose of 0.3 gram per kilo., causing in the frog convulsions, paralysis, and death from failure of respiration. Injected into the jugular vein of the dog, it acts on the vaso-motor and respiratory centers, and on the cardio-inhibitory centers in the medulla oblongata, causing the blood pressure to fluctuate below and above the normal, checking the respiration, and slowing the pulse. Successive doses, amounting to 1.5 grams per kilo., permanently lower the blood pressure, and cause death from respiratory paralysis. Small doses of *acetamide* injected into the dog raise the blood pressure, increase respiration rate, and diminish the pulse rate by the action of the cardio-inhibitory centers. Large doses (up to 5 grams per kilo.) may be injected with immunity, but these, after a time, cause extreme drowsiness and sleep. The effect of *propionamide* in the dog is small ; doses of 0.25 gram per kilo. strengthen the beat of the heart, lower the blood pressure, and act on the cardio-inhibitory centers, checking the pulse. *Benzamide* diminishes sensibility and reflex and voluntary activity in the frog. The fatal dose is 1.0 gram per kilo. Administered to the dog, it produces the additional effects of staggering, salivation, lowered temperature (1-2°), quickened respiration, and lessened pulse rate. Death results from failure of respiration. Injected into the jugular vein, it causes a rapid fall and rise in the blood pressure, but after repeated doses the pressure does not recover, and the heart is paralysed. The heart beat is lessened in force, the pulse becomes more frequent, and the respiration fails; at the same time, the pupils become dilated and sensibility is destroyed. The fatal dose is 0.5 gram per kilo. *Oxamide*, *thio-carbamide*, and *pyro-mucamide* have no appreciable effect on the dog.

*Anilides.*—Formanilide administered to the dog by the mouth or subcutaneously in doses of 0.4 gram per kilo., acts on the respiratory centers, quickening the respiration; on the cardio-inhibitory centers and heart, successively raising and lowering the pulse rate; and on the vaso-motor centers, lowering the blood pressure; it lowers the temperature, increases reflex activity, and produces feebleness and coma, and death from respiratory failure. *Acetanilide* given to the dog causes vomiting, weakness, lowering of temperature, quickening of pulse, darkening of blood, due to formation of methemoglobin, and cessation of respiration. When injected into the circulation, it acts on the heart, reducing the blood pressure and successively quickening and slowing the pulse. The rate of respiration at first increases, but ultimately diminishes and fails, causing death. The fatal dose is 0.3 gram per kilo. *Benzanilide* injected into the jugular vein of the dog at first increases the blood pressure, but after the administration of several doses diminishes it. It also quickens the respiration and lowers the temperature. It is probable that the action of the drug is due to mechanical clogging of the capillaries of the nerve centers by the deposition of fine crystals, since enormous doses may be taken into the stomach without any appreciable effect.

---

### PEPTOTOXIN.

E. SALKOWSKI (*Virchow's Archiv*, 124, 409; *Jour. Chem. Soc.*, 60, 1267). As a result of experiments on the digestion of proteids by means of pepsin, no poisonous substance soluble in water and amyl alcohol was formed, such as Brieger states he isolated from digested fibrin ; in other words, a "peptotoxin" in Brieger's sense of alkaloidal substance does not exist.

If putrefaction is prevented, leucine and tyrosine are also never formed in gastric digestion.

If putrefaction be allowed to occur, poisonous basic products make their appearance. Some specimens of meat contain poisonous bases previous to the occurrence of digestion at all. The resinous substance present in commercial amyl alcohol is also toxic. These facts, no doubt, explain Brieger's error.

That albumoses and peptones are toxic, none will deny ; the object of the present research is to show that their toxicity is one of their inherent properties and not due to any basic product or toxine separable from them.

